# Barriers and Facilitators Affecting Access to Health Care for People With Syphilis: Protocol for a Scoping Review

**DOI:** 10.2196/63561

**Published:** 2024-11-15

**Authors:** Rafaela Bezerra Façanha Correia, Rafaela Prudlik Mourad, Janmilli da Costa Dantas, Richardson Augusto Rosendo da Silva

**Affiliations:** 1 Department of Speech, Language and Hearing Disorders Federal University of Rio Grande do Norte Natal Brazil; 2 Department of Nursing Federal University of Rio Grande do Norte Natal Brazil; 3 Department of Nursing Faculty of Health Sciences of Trairi Federal University of Rio Grande do Norte Santa Cruz Brazil

**Keywords:** syphilis, acquired syphilis, congenital syphilis, gestational syphilis, Treponema pallidum, health services accessibility, access to health services, scoping review

## Abstract

**Background:**

Syphilis is a systemic, preventable, and curable infection caused by the bacterium *Treponema pallidum*. Despite being treatable, syphilis continues to have a high incidence, with a resurgence observed even in countries with strong health surveillance systems. This highlights the need to understand the various strategies used globally to improve access to care for individuals with syphilis.

**Objective:**

This scoping review aims to identify and map the barriers and facilitators affecting access to health care for people with syphilis.

**Methods:**

This scoping review will follow the methodology outlined by the Joanna Briggs Institute. The search will be conducted across several databases, including PubMed/MEDLINE, Scopus, Embase, LILACS (Virtual Health Library), and CINAHL (EBSCO). In addition, sources of unpublished studies or gray literature will be explored. Studies focusing on access to health care for individuals with syphilis will be included, regardless of geographic location, country, or language. Two independent reviewers will assess the results, and data will be extracted using a tool specifically developed for this review. The extracted quantitative data will be presented in tables and analyzed using descending hierarchical classification, represented by a class dendrogram. Barriers and facilitators will be categorized into dimensions of access.

**Results:**

Database searching began in October 2024. Full-text screening and review are expected to be completed in December 2024. Data extraction and analysis are expected to be completed by February 2025, and the final report will be completed in March 2025.

**Conclusions:**

The findings of this scoping review, guided by this protocol, will elucidate the main barriers and facilitators that affect access to syphilis treatment. This study may contribute to the practices of health professionals, managers, and the academic community, and provide relevant information for the population.

**Trial Registration:**

Open Science Framework Registries osf.io/kpsab; https://osf.io/kpsab

**International Registered Report Identifier (IRRID):**

PRR1-10.2196/63561

## Introduction

Syphilis is a systemic, preventable, and curable infection caused by the bacterium *Treponema pallidum*. The main route of transmission is sexual, but it can also be transmitted from mother to fetus during the gestational period [1]. If not treated, syphilis may cause serious complications, such as an increased risk of contracting other sexually transmitted infections, neurological and ocular changes, hearing or balance impairments (or both), death, neonatal death, prematurity, low birth weight, and stillbirths. Syphilis is the second most common cause of stillbirths due to infectious diseases worldwide [2].

Syphilis is one of the most important sexually transmitted infections in the world [3]. Studies estimate that more than 11 million new cases of syphilis occur annually worldwide, with high incidence in Latin America, Africa, and Asia [4]. Furthermore, some countries with good surveillance of sexually transmitted infections, such as the United States, Canada, Australia, and Japan, have reported a resurgence of syphilis after the COVID-19 pandemic [5].

The resurgence and high incidence of syphilis indicate a failure in its control and should be seen as an urgent call for targeted and intense action. Effective control requires sufficient funding, coordinated public policies on prenatal and sexual health care, clear guidelines, campaigns to encourage people to seek care without fear of discrimination, and educational programs. Thus, sustained and effective efforts are needed to control this infection [6]. The limited access to health care caused by social, structural, and economic barriers hinders the early detection and treatment of syphilis, resulting in perpetuated transmission and increased morbidity and mortality [7,8]. It is understood that caring for people with syphilis requires a multifaceted approach that involves collaboration between governments, managers, health professionals, and society [9].

It is necessary to know the different strategies adopted to care for people with syphilis worldwide. Currently, there are studies that point out some barriers or facilitators affecting access to care for people with syphilis; however, they address only a certain service or a classification of syphilis (acquired, gestational, or congenital) in a certain location or country [10-12], justifying this study. In addition, these barriers and facilitators may change over the years and may also be different in different countries, as they have different socioeconomic, cultural, and geographical contexts, as well as varied health systems, which is an additional part of this research.

In May 2024, a preliminary search was conducted in PubMed, Joanna Briggs Institute (JBI) Evidence Synthesis, PROSPERO, and the Open Science Framework. No other planned or ongoing reviews on this topic were identified.

Therefore, this scoping review aims to identify and map the barriers and facilitators affecting access to health care for people with syphilis. The results of this review will provide subsidies for discussions on public health policies aimed at the population with syphilis, the implementation of new projects, and the encouragement of research based on the gaps identified during the research. They will also allow health professionals to make more effective interventions, demonstrating the relevance of this research.

## Methods

In line with open science, this protocol is methodologically organized. It allows the entire scientific method to be replicated, thereby mitigating the risk of bias in research [13].

### Study Design

The proposed scoping review will be conducted in accordance with the JBI methodology for scoping reviews [14], based on the theoretical framework proposed by Arksey and O’Malley [15], and updated by Levac et al [16]. The PRISMA-ScR (Preferred Reporting Items for Systematic Reviews and Meta-Analyses Extension for Scoping Reviews) [17] will be used to report the results (Multimedia Appendix 1).

The stages of the scoping review include (1) definition and alignment of the objective with the research question; (2) development and alignment of inclusion criteria; (3) description of the search for evidence, selection and extraction of data, and presentation of evidence; (4) search for evidence; (5) selection of evidence; (6) extraction of evidence; (7) analysis of evidence; (8) presentation of the results; and (9) summary of evidence, conclusions, and implications of findings [14].

### Stage 1: Definition and Alignment of the Objective With the Research Question

We used the population, concept, and context mnemonic to assist in forming the research question and developing the study title. Here, we define the population as individuals with syphilis, the concept as access to health care, and the context as the environment of health services globally. Therefore, the question that will guide this study is as follows: What are the barriers and facilitators affecting access to health care for people with syphilis?

### Stage 2: Development and Alignment of Inclusion Criteria

#### Overview

Studies will be included if the full text is available. Studies published in any language will be included. Those in a language other than Portuguese will initially be translated using Google Translate, and the full text will be professionally translated if the study meets the inclusion criteria. This scoping review will consider gray literature, such as theses, dissertations, and government reports. Individual case reports, clinical protocols, conference and event summaries, and journal editorials will be excluded as their usefulness in this review is not anticipated. Considering the mnemonic adopted, the eligible studies are the following.

#### Population

This review will consider studies that address people with acquired, congenital, or gestational syphilis. Acquired syphilis is conceptualized as a disease transmitted from one person infected with the bacterium *T pallidum* to another during sex (anal, vaginal, or oral) without a condom or by blood transfusion. Particularly when it affects a pregnant woman, it is called gestational syphilis [18].

#### Concept

This study will consider conceptual studies that discuss access to health care. Access has been conceptualized in numerous ways. Here, we have defined access as the opportunity to identify health care needs; seek health care services; reach, obtain, or use health care services; and actually have health needs resolved [19].

#### Context

The context of interest is health services. Thus, studies conducted in primary care units, outpatient departments, multidisciplinary or specialized clinics (or both), maternity wards, hospitals, and emergency care units will be included. In addition, any geographic scenario and country will be considered.

### Stage 3: Description of the Search for Evidence, Selection, Extraction of Data, and Presentation of Evidence

An initial search limited to PubMed and the Virtual Health Library (*Biblioteca Virtual en Salud*; VHL) was conducted to identify the main Medical Subject Headings (MeSH) terms and Health Sciences Descriptors (Descriptors in Health Sciences; *Descritores em Ciências da Saúde* [DeCS]) related to the topic. The search strategy was constructed using controlled health vocabulary, including MeSH, DeCS, and Emtree, to expand results across different databases. Then, a search was conducted to identify synonyms and keywords. The search strategy was expanded and validated by an experienced librarian (AA). Multimedia Appendix 2 shows the complete search strategy built for PubMed/MEDLINE.

The search strategy will be adapted for each database and information source, including PubMed/MEDLINE, Scopus, Embase, LILACS via VHL (in Portuguese, Spanish, and English), and CINAHL (EBSCO). These databases were selected to allow for a more comprehensive search. In addition, sources of unpublished studies or gray literature will be searched, including Google Scholar (limited to the first 100 results), Brazilian Digital Library of Theses and Dissertations, the Catalog of Theses and Dissertations of the Coordenação de Aperfeiçoamento de Pessoal de Nível Superior (CAPES), and ProQuest Dissertations and Theses Global.

Reference lists from all included sources will be reviewed to identify any additional relevant studies. If necessary, the authors of the included studies will be consulted by email for any additional information.

### Stage 4: Search for Evidence

After the search, all identified quotes will be collected and imported into the free version of Rayyan (Qatar Foundation), where duplicates will be removed and studies will be selected.

Two reviewers (RB and RP) will conduct a pilot test to reduce bias, standardize the selection process, and verify compliance with the study protocol. The pilot test will be carried out using a random sample of 25 titles and abstracts, following the eligibility criteria. The team will discuss any discrepancies and change criteria and definitions, if necessary. Screening will begin only after at least 75% agreement [14].

Once the pilot test is complete, the 2 reviewers (RB and RP) will use Rayyan to read the titles and abstracts of all studies identified by the eligibility criteria. If there are disagreements between reviewers during the process, resolution will be reached through consultation with a third reviewer (JC).

### Stage 5: Selection of Evidence

After reading the titles and abstracts of all studies, potentially relevant sources will be obtained in full and exported to Google Drive. The full text of selected studies will undergo a detailed assessment against the inclusion criteria by 2 independent reviewers (RB and RP). Reasons for exclusion will be recorded. In case of disagreements between reviewers during the selection process, resolution will be reached by consulting the third reviewer (JC). The study selection process will be reported in the PRISMA-SCR flowchart [20].

### Stage 6: Extraction of Evidence

The reviewers developed a draft data extraction form (Table 1), based on suggestions provided in the JBI methodology for scoping reviews [14]. For better data extraction, the framework proposed by Levesque et al [19] outlines 5 dimensions of accessibility: approachability, acceptability, availability and accommodation, affordability, and appropriateness.

Approachability refers to whether individuals with health needs can identify available services, understand their potential impact, and access them. This dimension is influenced by factors such as transparency, information about available treatments and services, outreach activities, health literacy, knowledge about health, and cultural beliefs. Acceptability involves cultural and social factors that determine whether individuals find the aspects of a service (eg, the gender or social group of providers, the associated medical beliefs) appropriate and acceptable [19].

Availability and accommodation pertain to whether health services are physically accessible and available in a timely manner. This includes the existence of adequate facilities, building accessibility, transportation systems, flexible working hours, and the qualifications of health professionals. Affordability relates to the economic capacity of individuals to spend resources and time on necessary services. Factors such as poverty, social isolation, and indebtedness can limit an individual’s ability to pay for needed care. Appropriateness concerns the suitability and quality of the services provided, including their integrated and continuous nature [21].

**Table 1:** Preliminary data extraction form.

Variable	Standardization
First author and year of publication	Identify the first author and year of publication of the study
Objective	Detail the objective of the study
Study design	Classify according to modality (theoretical, field, and bibliographic), objectives (exploratory, descriptive, and explanatory), and approach (quantitative and qualitative)
Study population	Detail the population of the study
Data collection procedure	Describe the type of data collection procedure used in the study
The country where the research was carried out	Identify the country where the study was conducted
Syphilis classification	Identify the classification of syphilis covered in the study: congenital syphilis, gestational syphilis, or acquired syphilis
Study location	Identify in which service or institution the study was carried out (basic health units, outpatient clinics, multidisciplinary or specialized clinics, maternity wards, hospitals, and emergency care units)
Results	Describe the main barriers and facilitators to access health care for people with syphilis based on the 5 dimensions of accessibility (Approachability, Acceptability, Availability and accommodation, Affordability, and Appropriateness) and 5 corresponding abilities of populations (Ability to perceive, Ability to seek, Ability to reach, Ability to pay, and Ability to engage) [19] The barriers and facilitators that are explicit in the texts will be extracted

Initially, there will be a pilot test for the use of the extraction tool. Two reviewers (RB and RP) will conduct the test using a sample of 10 papers. The goal is to ensure that important information is completely extracted and that the process is standardized. At this stage, relevant variables that emerged during the test may be included.

Two independent reviewers (RB and RP) will use the form to extract data from all eligible studies. In case of discrepancies between reviewers, a consensus will be reached through discussion with the third reviewer (JC). The authors of the studies will be contacted to obtain missing or additional data, if needed.

### Stage 7: Analysis of Evidence

The results will be analyzed quantitatively and qualitatively. Quantitative analysis will use descriptive statistics with absolute and relative frequency. The Thematic Content Analysis method will be used for qualitative analysis. This method uses systematic and objective procedures, consisting of unraveling the “nuclei of meaning” that decompose communication and whose presence or frequency of appearance can signify something relevant to the chosen analytical objective [22]. The IRaMuTeQ software, developed by Pierre Ratinaud, will contribute to the lexical analysis of the textual corpus [23].

### Stage 8: Presentation of Results

Results will be presented using a PRISMA-ScR flowchart [20]. The extracted quantitative data will be presented in tables and by the class dendrogram for descending hierarchical classification. This allows the analysis of text segments that present similar vocabulary and vocabulary different from other text segments, simultaneously calculating distances and proximities based on chi-square tests. With these analyses, IRaMuTeQ organizes the words in a dendrogram—which represents the quantity and linguistic composition of classes based on a grouping of terms—from which the absolute frequency of each of them and the aggregate chi-square value are obtained [23].

The barriers and facilitators will be categorized into dimensions of access: (1) Accessibility, (2) Acceptability, (3) Availability and accommodation, (4) Affordability, and (5) Appropriate. From each thematic category, there will be a summary description of the extracted information.

### Stage 9: Summary of Evidence, Conclusions, and Implications of Findings

Once the previous steps have been completed, a summary of the evidence will be drawn up to support the conclusion. We will list the gaps in knowledge to guide future research.

## Results

The database search began in October 2024, generating 995 articles, of which 306 were duplicates. The next step will be to search the gray literature. Upon completion, there will be screening of titles and abstracts and review of the full text, with completion scheduled for December 2024. Data extraction and analysis should be carried out in January and February 2025, with the expectation that the final report will be ready for shipping by March 2025. The results of this research will be published in open-access and peer-reviewed journals, a testament to our commitment to disseminating knowledge in the scientific community and ensuring the credibility of our findings.

## Discussion

### Overview

The scoping review has gained prominence globally in the field of health evidence synthesis [24]. This methodology was chosen for this study because it aims to map the literature in a specific area of interest—in this case, access to health services for people with syphilis on a global scale. The scoping review is particularly useful when reviews on a subject have not yet been published. It allows for the inclusion of a broad range of study designs and aims to identify and synthesize the available evidence [14,15].

We chose to use IRaMuTeQ for data analysis, a free software that enables various types of textual data analysis, from basic lexicography (eg, word frequency calculation) to more complex multivariate analyses, such as descending hierarchical classification and similarity analysis [23]. The software organizes the distribution of vocabulary in an easily understandable and visually clear manner, such as through similitude analysis and word clouds. The advantages of using software for data analysis include improved organization and separation of information, increased process efficiency, easier location of text segments, and faster coding compared with manual methods [25].

A key strength of this scoping review is the experienced research team, who are well versed in the study of syphilis and in applying the scoping review methodology. To ensure a highly sensitive search strategy for this protocol, we collaborated with a librarian affiliated with the researchers institution, which enhanced the comprehensiveness of the search and allowed for greater access to relevant literature. Importantly, the research will not be limited by time or language constraints.

Access to health services has been a subject of analysis since the latter half of the 20th century [26]. Over the years, authors have conceptualized access to health care differently. The Levesque, Harris, and Russell model was chosen for its comprehensive scope. Their conceptual framework, published almost 7 years ago, is still considered relatively new. One key aspect of this model is its portrayal of access as a dynamic process or journey in contrast to the static concept as defined by others. The 5 proposed dimensions are interconnected, offering a multidimensional view of access to health within the context of health systems, thereby shedding light on the complexity of the topic [19,27].

The most common challenges of this model are related to repeated instances, and there may be difficulties in categorizing information into specific dimensions or when responses fall into more than 1 dimension. Another weakness is the inability to consider time-related access elements (waiting time and travel time) [27].

Access to health care for people with syphilis requires ongoing attention from health managers, professionals, and researchers due to the high incidence of the infection in various regions globally. Numerous initiatives have been developed in recent years to combat this epidemic. For instance, in 2016, the World Health Organization launched the Global Health Sector Strategy on Sexually Transmitted Infections 2016-2021, aiming for a 90% reduction in the incidence of *T pallidum* and fewer than 50 cases of congenital syphilis per 100,000 live births in 80% of countries by 2023 [28]. In 2017, the Pan American Health Organization set goals to increase syphilis screening and appropriate treatment coverage in pregnant women to 95% or more, aiming to eliminate vertical transmission of syphilis [29].

Despite these governmental efforts, syphilis remains a persistent public health challenge. Watt et al [30] identified several factors that contribute to low access among young people to sexual and reproductive health services, including a lack of knowledge about available services, cultural and religious barriers, judgmental attitudes of health professionals, and insufficient privacy in health facilities.

Additional barriers to syphilis testing and care include stigma, discrimination, gender-based violence, past negative health care experiences, reluctance to disclose sexual practices, confidentiality concerns, and limited access to health facilities [31,32]. These barriers can be addressed through continuing education for health care teams, which fosters reflection on the work process and recognizes the needs and potential of the service. In-service learning is essential for improving the quality of health care.

Socioeconomic disparities, limitations in government financial transfers for health in specific regions, the concentration of health services in urban areas, and restricted access for populations in rural and riverside areas also hinder care for patients with syphilis. Understanding these barriers and facilitators allows health policy makers and managers to act at local or regional levels, designing policies and programs based on successful experiences identified in the scoping review, with adaptations for the specific context.

As identified through this scoping review, there is a significant knowledge gap regarding the barriers and facilitators to accessing health care for people with syphilis, whether acquired, congenital, or gestational, in a global context.

The scarcity of studies on these topics remains an obstacle to controlling syphilis at the community level. This study aims to explore the breadth of the literature, map current evidence, and identify gaps in knowledge about syphilis worldwide.

Moreover, it is anticipated that the results of this scoping review will be significant in prompting changes at both macropolitical and micropolitical levels, leading to better access to health care services and improved quality of care for people with syphilis.

The study also aims to raise awareness among society and health professionals about the importance of providing welcoming and attentive care for people with syphilis, ensuring that they receive the same rights to quality care as anyone else.

### Limitations

The use of descriptors and search terms in only English, Portuguese, and Spanish may limit the study by potentially excluding relevant research published in other languages. In addition, the absence of searches on institutional websites across all countries for gray literature may also be a limitation, although this is not expected to significantly impact the development of the scoping review.

Furthermore, the quality and methodological rigor of the included studies will not be assessed, as this is characteristic of a scoping review, which could introduce biases in the synthesis of the results.

### Conclusions

This paper presents a scoping review protocol. Upon completion of the study, it will map the barriers and facilitators to health care access for people with syphilis, identify challenges to health care access in various parts of the world, and elucidate potential strategies for improving service delivery in syphilis care.

The full results will be disseminated through scientific conferences and international publications, with the aim of reaching 3 key target audiences: public health policy makers, health care professionals who care for people with syphilis, and researchers focused on this topic.

Ultimately, the study may highlight the need to develop and refine policies that prevent barriers to care from impeding access to health services, particularly in Brazil.

## Appendix

Multimedia Appendix 1 PRISMA-P (Preferred Reporting Items for Systematic review and Meta-Analysis Protocols) 2015 checklist.

Multimedia Appendix 2 Search terms and results from an initial search conducted in PubMed.

## Abbreviations

CAPES: Coordenação de Aperfeiçoamento de Pessoal de Nível Superior
DeCS: Health Sciences Descriptors (Descritores em Ciências da Saúde)
JBI: Joanna Briggs Institute
MeSH: Medical Subject Headings
PRISMA-ScR: Preferred Reporting Items for Systematic Reviews and Meta-Analyses Extension for Scoping Reviews
VHL: Virtual Health Library (Biblioteca Virtual en Salud)
