# Supplementary material for: Barriers and Facilitators Affecting Access to Health Care for People With Syphilis: Protocol for a Scoping Review
Source: JMIR Res Protoc. 2024 Nov 15;13:e63561. doi: 10.2196/63561 (PMC11607569; doi:10.2196/63561)
Supplement: Multimedia Appendix 2 [file resprot_v13i1e63561_app2.doc]

**Multimedia Appendix 2.** Complete strategy for searching Medline/PubMed.

| **Search** | **Query PubMed**  **(05/05/2024)** | **Records recovered** |
| --- | --- | --- |
| #1 | ("Treponema pallidum"[MeSH Terms] OR "Treponema pallidum"[All Fields] OR "Syphilis"[MeSH Terms] OR "Syphilis"[All Fields] OR "syphilis, congenital"[MeSH Terms] OR "syphilis congenital"[All Fields] OR "Syphilis in Pregnancy"[All Fields] OR "Gestational Syphilis"[All Fields] OR "acquired syphilis"[All Fields]) | 43.314 |
| #2 | ("Health Services Accessibility"[MeSH Terms] OR "Health Services Accessibility"[All Fields] OR "Access to Health Services"[All Fields] OR "Access to Health Care"[All Fields] OR "Access to Care"[All Fields] OR "Access to Cares"[All Fields] OR "Access to Medications"[All Fields] OR "Access to Medication"[All Fields] OR "Access to Treatment"[All Fields] OR "Access to Treatments"[All Fields] OR "health care access"[All Fields]) | 163.439 |
| #1 AND #2 | ("Treponema pallidum"[MeSH Terms] OR "Treponema pallidum"[All Fields] OR "Syphilis"[MeSH Terms] OR "Syphilis"[All Fields] OR "syphilis, congenital"[MeSH Terms] OR "syphilis congenital"[All Fields] OR "Syphilis in Pregnancy"[All Fields] OR "Gestational Syphilis"[All Fields] OR "acquired syphilis"[All Fields]) AND ("Health Services Accessibility"[MeSH Terms] OR "Health Services Accessibility"[All Fields] OR "Access to Health Services"[All Fields] OR "Access to Health Care"[All Fields] OR "Access to Care"[All Fields] OR "Access to Cares"[All Fields] OR "Access to Medications"[All Fields] OR "Access to Medication"[All Fields] OR "Access to Treatment"[All Fields] OR "Access to Treatments"[All Fields] OR "health care access"[All Fields]) | 236 |
